# Immunology of a unique biological structure: the *Echinococcus* laminated layer

**DOI:** 10.1093/procel/pwac023

**Published:** 2022-07-15

**Authors:** Álvaro Díaz, Anabella A Barrios, Leticia Grezzi, Camila Mouhape, Stephen J Jenkins, Judith E Allen, Cecilia Casaravilla

**Affiliations:** Área Inmunología, Departamento de Biociencias (Facultad de Química) and Cátedra de Inmunología, Instituto de Química Biológica (Facultad de Ciencias), Universidad de la República, Montevideo, Uruguay; Área Inmunología, Departamento de Biociencias (Facultad de Química) and Cátedra de Inmunología, Instituto de Química Biológica (Facultad de Ciencias), Universidad de la República, Montevideo, Uruguay; Área Inmunología, Departamento de Biociencias (Facultad de Química) and Cátedra de Inmunología, Instituto de Química Biológica (Facultad de Ciencias), Universidad de la República, Montevideo, Uruguay; Área Inmunología, Departamento de Biociencias (Facultad de Química) and Cátedra de Inmunología, Instituto de Química Biológica (Facultad de Ciencias), Universidad de la República, Montevideo, Uruguay; Centre for Inflammation Research, Queen’s Medical Research Institute, University of Edinburgh, Edinburgh, EH8 9JU, UK; Lydia Becker Institute of Immunology and Inflammation, School of Biological Sciences, University of Manchester, Manchester Academic Health Sciences Centre, Manchester, M13 9NQ, UK; Área Inmunología, Departamento de Biociencias (Facultad de Química) and Cátedra de Inmunología, Instituto de Química Biológica (Facultad de Ciencias), Universidad de la República, Montevideo, Uruguay

**Keywords:** mucin, complement, *Echinococcus*, macrophage, dendritic cell, Clec4F

## Abstract

The larval stages of the cestode parasites belonging to the genus *Echinococcus* grow within internal organs of humans and a range of animal species. The resulting diseases, collectively termed echinococcoses, include major neglected tropical diseases of humans and livestock. *Echinococcus* larvae are outwardly protected by the laminated layer (LL), an acellular structure that is unique to this genus. The LL is based on a fibrillar meshwork made up of mucins, which are decorated by galactose-rich *O*-glycans. In addition, in the species cluster termed *E. granulosus sensu lato*, the LL features nano-deposits of the calcium salt of *myo*-inositol hexakisphosphate (Ins*p*_6_). The main purpose of our article is to update the immunobiology of the LL. Major recent advances in this area are (i) the demonstration of LL “debris” at the infection site and draining lymph nodes, (ii) the characterization of the decoy activity of calcium Ins*p*_6_ with respect to complement, (iii) the evidence that the LL mucin carbohydrates interact specifically with a lectin receptor expressed in Kupffer cells (Clec4F), and (iv) the characterization of what appear to be receptor-independent effects of LL particles on dendritic cells and macrophages. Much information is missing on the immunology of this intriguing structure: we discuss gaps in knowledge and propose possible avenues for research.

## Introduction

The laminated layer (LL) is the crucial structure at the host-parasite interface in the parasitic diseases collectively known as echinococcoses. We reviewed the biology of the LL including structural aspects in depth in 2011 and 2015 (Díaz et al., 2011a, 2011b, 2015). For most of the present article, we therefore focus on the immunological side, on which there is significant recent information. Our discussion makes emphasis on the interactions of LL with host complement, antibodies, dendritic cells, and macrophages, but it does touch on additional aspects of immunology.

## A primer on *Echinococcus* and the echinococcoses

**Figure 1.**
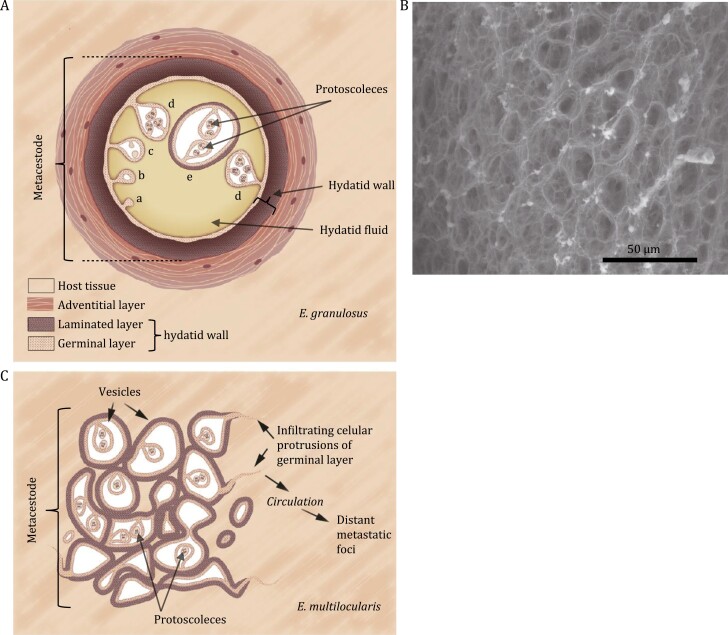
Structure of *Echinococcus* metacestodes and the LL. General structure of the *E. granulosus* (A) and *E. mutilocularis* metacestodes (B). In (A), a–d represent stages in the development of protoscoleces. Note that the “adventitial layer” often surrounding *E. granulosus* metacestodes is the fibrous host reaction, and that metacestode plus adventitial layer form the so called “hydatid cyst”. Reproduced and modified with permission from reference (Thompson, 2017) (published by Elsevier). (C) Appearance of the *E. granulosus* LL under the scanning electron microscope, using critical point drying. The image corresponds to material from a natural infection in cattle, and was obtained by Prof. Aline Miller and Ziqing Zhou (Department of Chemical Engineering, Manchester Institute of Biotechnology, University of Manchester, UK).

*Echinococcus* is a genus of taeniid cestode platyhelminths (tapeworms). The larval stages of the species within this genus lodge in internal organs of mammals including humans, causing the diseases collectively known as echinococcoses (Brunetti and White, 2012; Thompson and Jenkins, 2014; Thompson, 2017; Wen et al., 2019; Lightowlers et al., 2021). Within this disease complex, cystic echinococcosis (CE; also known as hydatid disease) has the broadest geographical distribution, and it is considered a neglected tropical disease (Agudelo Higuita et al., 2016). CE is caused by a cluster of species that can be discriminated only by molecular means, and that have mainly ungulates as the natural hosts of their larval stages. This cluster, termed *E. granulosus sensu lato,* encompasses *E. granulosus sensu stricto* and four other species (Lymbery, 2017). Because of lack of full species information in many cases, throughout this article, “*E. granulosus*” refers to *E. granulosus sensu lato* unless otherwise specified. The larval *E. granulosus* parasite (metacestode) is a fluid-filled, bladder like structure known as hydatid, which can attain tens of cm in diameter in the mammalian host (Thompson, 2017) (Fig. 1A). CE can affect virtually any internal organ, but liver, and lungs are the most commonly affected. The other important echinococcosis is alveolar echinococcosis (AE), caused by *E. multilocularis*. In this case, the metacestode forms a series of interconnected tubules and chambers that invades the host’s liver (Fig. 1B). Natural hosts are wild rodents, and human infection is life-threatening if untreated (Brunetti and White, 2012; Thompson, 2017; Casulli et al., 2019; Lightowlers et al., 2021).

Echinococcosis normally results from the ingestion of *Echinococcus* eggs, passed out with the feces of a canid host that harbors the adult tapeworm. After their ingestion, eggs release oncospheres that traverse the gut wall and are carried via blood or lymph to solid organs where metacestodes develop (Thompson, 2017). In addition to the described (primary) form of infection, echinococcosis can also develop after infection by protoscoleces, called “secondary infection”. Protoscoleces (Fig. 1A and 1B) are parasite forms normally destined to infect the primary host but are capable of reverse development into metacestodes when released into mammalian tissues (Thompson, 2017). In addition to being often employed experimentally for infecting mice, this secondary infection route causes natural infections when protoscoleces escape from the metacestode, as it can happen during surgery.

Irrespective of species differences, *Echinococcus* larvae develop as fluid-filled chambers bounded by a thin layer of live parasite tissue, called the germinal layer (Fig. 1A and 1B). The acellular LL is synthesized directionally by germinal layer towards the host; germinal layer plus LL together form what is known as the hydatid wall (especially in CE). While the *E. multilocularis* LL usually has tens of µm thickness, in *E. granulosus* it reaches hundreds of µm to over a mm in thickness (Reinehr et al., 2020). The LL is deployed 14–20 days post infection after primary infections, and in approximately twice this time after secondary infections (Díaz et al., 2011a). The LL precludes direct contact between the germinal layer and host immune cells, thus affording parasite cells partial protection against immune effectors (Gottstein et al., 2002; Díaz et al., 2011b).

The systemic immune responses to larval *Echinococcus* are, as can be expected of responses to helminths, biased towards Th2, although Th1, as well as Th17, components are also detected (Zhang et al., 2012; Díaz, 2017; Gottstein et al., 2017; Díaz et al., 2018; Wang et al., 2018; Yasen et al., 2021). As should also be expected of parasites capable of long-lived infections, *Echinococcus* larvae elicit strong regulatory responses in the host, in which the immune-suppressive cytokine TGF-β and Foxp3^+^ regulatory T cells appear to be central (Mejri et al., 2011; Pang et al., 2014a, 2014b, 2018; Gottstein et al., 2017; Díaz et al., 2018; Wang et al., 2018; Fratini et al., 2020). Accordingly, the local response to the metacestode is usually remarkably subdued. However, mismatch between host and parasite species (e.g. cattle or pig infection by *E. granulosus sensu stricto*) results in a granulomatous-type local inflammatory response, which is damaging to the parasite (Díaz et al., 2000, 2018; Sakamoto and Cabrera, 2003; Basika et al., 2012; Riesle et al., 2014; Thompson, 2017).

## A summary of the LL structure

The fabric of the LL is a meshwork of mucins (Casaravilla and Díaz, 2010; Hulsmeier et al., 2010; Díaz et al., 2011a), i.e. non-globular polypeptide backbones (apomucins) decorated by multiple (mucin-type) *O*-glycans. Animal mucins usually form loose, highly hydrated aggregates, like in mucus (Thornton et al., 2018). The LL mucins also form a highly hydrated gel-like structure (Irigoín et al., 2004; Díaz et al., 2011a). However, different from mucus, the LL mucin meshwork forms a physically coherent elastic structure, which in the case of *E. granulosus* allows the hydatid to be turgid. Under the transmission electron microscope the mucin fabric appears as a disorganized meshwork of fine fibrils (Morseth, 1967; Sakamoto and Sugimura, 1969; Richards et al., 1983). By scanning electron microscopy using critical point drying, the *E. granulosus* appears sponge-like (Fig. 1C).

The sequences of the LL apomucins have been deduced from genomic and transcriptomic data, but without any proteomic confirmation to date (Díaz et al., 2011a, 2015; Parkinson et al., 2012; Tsai et al., 2013; Zheng et al., 2013). These sequences code for (mostly short) secreted apomucins, each comprising a short non-glycosylated N-terminal extension, a mucin domain with a high predicted density of *O*-glycosylation, and a C-terminal signal for the incorporation of a glycosylphosphatidylinositol anchor (Parkinson et al., 2012; Tsai et al., 2013; Zheng et al., 2013); more details are given in Díaz et al., (2015).

The glycans decorating the LL mucins have been elucidated for *E. granulosus* and *E. multilocularis* (Hülsmeier et al., 2002; Díaz et al., 2009, 2015; Hulsmeier et al., 2010; Lin et al., 2013; del Puerto et al., 2016). They are based on the conventional mucin *O*-glycan cores 1 and 2, and are elongated and capped mostly with Gal residues. Therefore, most of the di- or tri-saccharide motifs exposed for interaction with host lectins (carbohydrate-binding proteins) or antibodies feature either α- or β-Gal as terminal monosaccharide residues. The *E. multilocularis* glycans are less elongated than their *E. granulosus* counterparts, generating subtle differences in the Gal-rich motifs present (Díaz et al., 2015; del Puerto et al., 2016). *E. multilocularis* metacestodes in culture lose all viability in the presence of α- or β-galactosidases, suggesting that the intact LL glycans are not needed solely for protection from the host (Wang et al., 2021).

The *E. granulosus* LL is formed, in addition to the mucin meshwork, by the calcium salt of *myo*-inositol hexakisphosphate (Ins*p*_6_). Ins*p*_6_ is a ubiquitous nuclear and cytosolic metabolite in eukaryotes (Raboy, 2003); in addition, in vacuolar compartments in plant seeds, it forms solid deposits with various metal ions (Madsen and Brinch-Pedersen, 2020). Ins*p*_6_ is insoluble in the presence of several divalent and trivalent metal ions, including Ca^2+^ at the concentrations found in extracellular and vesicular system compartments in animals (Veiga et al., 2006). In *E. granulosus*, Ins*p*_6_ reaches a vesicular system compartment, precipitates as calcium salt, and is exocytosed from the GL onto the LL in the form of deposits measuring approximately 40 nm in diameter (Morseth, 1967; Richards et al., 1983; Irigoín et al., 2004). The deposits are easily detected in the LL by transmission electron microscopy, appearing as naturally electron-dense “granules” interspersed among the mucin fibrils (Morseth, 1967; Richards et al., 1983; Irigoín et al., 2004). The adaptation is not common to the genus *Echinococcus*, as it is absent, by chemical, and/or ultrastructural evidence, in *E. multilocularis* and *E. vogeli* (Ingold et al., 2001; Irigoín et al., 2002).

The LL owes its name to its concentric “laminations” observable by light microscopy or scanning electron microscopy at low magnification, in *E. granulosus* specially (Ingold et al., 2001; Díaz et al., 2011a; Reinehr et al., 2020). These “laminations” appear to result from different degrees of compaction of the mucin fibrils and calcium Ins*p*_6_ deposits (Morseth, 1967).

The *E. granulosus* LL is freely permeable to macromolecules up to the size of immunoglobulin G (150 kDa) at least (Coltorti and Varela-Díaz, 1974). Indirect evidence suggests some limitation to the permeation of larger molecules (750 kDa and above) (Breijo et al., 2008; Barrios et al., 2019). The calcium Ins*p*_6_ deposits represent a truly enormous surface available for adsorption of soluble host proteins (Casaravilla et al., 2006). In agreement, the bulk of the abundant host proteins found in the LL (other than immunoglobulins and terminal complement components) can be solubilized by calcium chelators (Díaz et al., 2000a, 2000b; Basika et al., 2012; Barrios et al., 2019) (our unpublished observations).

In sum, we have a basic understanding of LL structure, but major knowledge gaps remain. Among several aspects, proteomic data on the mucins would be very valuable, confirming the inferred apomucin sequences and allowing to explore their possible post-translational modifications (not restricted to glycosylation).

## Molecular immunological aspects: the LL and host complement

The *E. granulosus sensu lato* LL activates host complement only poorly (Irigoín et al., 1996; Ferreira et al., 2001). This surely reflects an adaptation to minimize complement-mediated inflammation, known to be detrimental to the parasite (Ferreira et al., 2000; Breijo et al., 2008). The most powerful pro-inflammatory peptide of complement arises from the cleavage of C5, i.e. in the first step of the terminal complement pathway (Merle et al., 2015a). Possibly in agreement, the control of complement activation on LL-derived materials is more evident at the terminal complement pathway level than at the level of activation of C3, the central component (Irigoín et al., 1996). C3 activation is a self-amplified process, and this is central to the alternative complement pathway. The major physiological regulator curtailing C3b amplification is factor H (Merle et al., 2015b). Factor H both displaces factor B from activated C3 (C3b) and acts as a cofactor for the proteolytic inactivation of C3b by factor I (Sánchez-Corral et al., 2018). C3b inactivation on the LL *in vitro* is verified to proceed rapidly on the mucins and even more rapidly on calcium Ins*p*_6_ (Irigoín et al., 2008; Barrios et al., 2019). The whole of the factor I-cofactor activity for C3b inactivation detected in hydatid wall extracts corresponds to factor H derived from the host, rather than any parasite-derived regulators (Díaz et al., 1997). Together, these data indicate that factor H has functionally relevant affinities for calcium Ins*p*_6_ and for certain (unidentified) sites on the mucins. Thus, amplification of C3 activation/deposition on the LL is avoided by offering binding sites for host factor H (Irigoín et al., 2008; Barrios et al., 2019).

**Figure 2.**
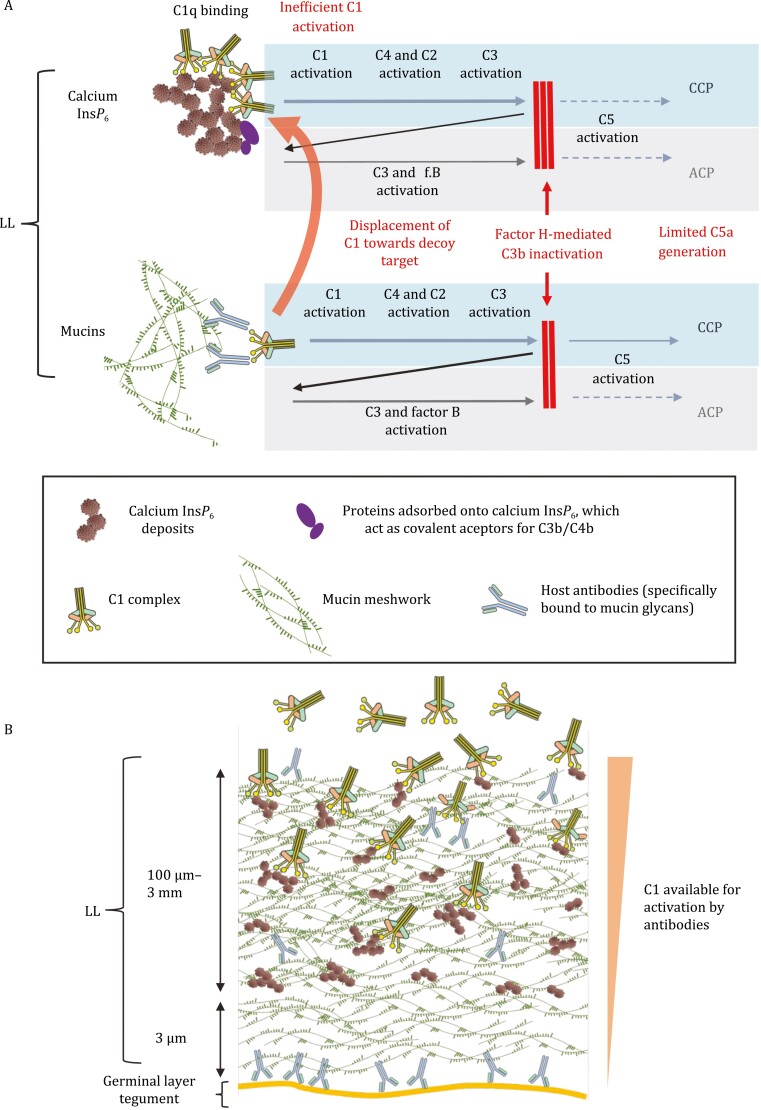
Proposed model summarizing complement activation and control on the LL and the hydatid wall. (A) Although the lectin pathway is not activated by the LL, the structure interacts both with the classical (CCP) and the alternative complement pathways (ACP). Calcium Ins*p*_6_ in the LL binds C1 complex through its C1q component. Although inefficiently, this causes C1 activation and initiates the CCP. This results in deposition of the activated forms of the CCP component C4 (C4b) and of the central component C3 (C3b) on calcium Ins*p*_6_ itself. As Ins*p*_6_ has no covalent acceptors for C4/C3, this deposition is bridged by adsorbed serum proteins. C3b deposited on calcium Ins*p*_6_ is very rapidly inactivated in factor H-dependent fashion, which also means that ACP activation is curtailed. C1 binding to antibodies bound to the mucins is reduced as a consequence of the abundant calcium Ins*p*_6_ acting as a decoy target for C1. Any remaining CCP activation on the mucins, as well as ACP activation, is curtailed as a consequence of factor H having affinity for (unknown sites in) the mucins. Overall, calcium Ins*p*_6_ re-directs CCP activation from the mucin-bound antibodies onto itself, without either enhancing or reducing C5 activation on the LL. (B) The high binding capacity of calcium Ins*p*_6_ deposits for C1q, combined with a restriction to the access of C1 imposed by the LL mucin gel would prevent the binding of functional C1 to the germinal layer tegument. This would avoid CCP activation triggered by antibodies bound to the germinal layer. In other words, the re-directioning of CCP activation onto calcium Ins*p*_6_, although “neutral” when considering C5 activation by the LL only, would have a parasite-protective role when the germinal layer is also considered. The previously reported absence of calcium Ins*p*_6_ deposits in the innermost 3 µm of the LL would prevent bystander damage to the germinal layer tegument by activation CCP initiated on calcium Ins*p*_6_. Part (B) has been reproduced from reference (Barrios et al., 2019) (published by Elsevier).

Complement control based on the prevention of C3b amplification could be overridden by strong activation of the lectin and/or classical pathways (Merle et al., 2015b). The LL does not activate the human lectin pathway *in vitro* (Barrios et al., 2019). However, as detailed in the following section, the LL mucins are targets of host antibodies, and part of these belong to classes or sub-classes that activate the classical pathway (Barrios et al., 2019). Antibodies bound to antigens initiate the classical pathway by binding (through their Fc portions) to C1q, the recognition subunit of the C1 complex (Merle et al., 2015b). Interestingly, most of the C1q-binding capacity of the LL is not due to antibodies but rather due to calcium Ins*p*_6_ (Barrios et al., 2019). However, C1 activation on calcium Ins*p*_6_ is inefficient, similar to what is observed on other polyanionic molecules that bind C1q (Garlatti et al., 2010; Barrios et al., 2019). Also, C3b deposited on calcium Ins*p*_6_ is inactivated particularly fast. In sum, the net effect of C1q binding to Ins*p*_6_ is probably to limit local availability of C1 for activation on antibodies, without making a contribution to the activation of C5 (Barrios et al., 2019). We envisage that this mechanism may be particularly important for the avoidance of classical pathway activation by antibodies bound to the surface of the germinal layer (Riesle et al., 2014), where such activation may damage the parasite through the lytic mechanism of complement. In this hypothesis, the absence of calcium Ins*p*_6_ in the 3 μm of LL closest to the germinal layer (Richards et al., 1983) may keep complement activation (however inefficient) away from the parasite cells. Our *in vitro* data also suggested that C1 complex permeation through the LL is restricted (Barrios et al., 2019). Thus, the *E. granulosus* LL probably minimizes C1 complex access to antibodies bound to the germinal layer through a combination of a sieve effect and the abundance of a decoy target for C1q (Fig. 2).

In sum, the *E. granulosus* LL appears to control antibody-dependent classical pathway activation by using calcium Ins*p*_6_ as a decoy, and alternative pathway activation/amplification by accumulating host factor H. In terms of perspectives, it would be of particular interest to analyze complement activation and control on the *E. multilocularis* LL, in view of absence of calcium Ins*p*_6_ in this species.

A subjective, chronological, account of the complex path that led us to find the calcium Ins*p*_6_ deposits and understand their interactions with complement is given in Box 1.

**Box 1. A convoluted research story: complement and calcium inositol hexakiphosphate deposits.**

This Box gives a subjective account of the discovery of calcium Ins*p*_6_ deposits in the *E. granulosus* LL, in the context of research initially aimed at understanding the regulation of host complement activation on the LL. Part of the motivation for this Box is to pay tribute to the memory of Robert B. (“Bob”) Sim, who passed away last year (Díaz, 2021). Bob was a world authority in complement biochemistry, who developed most of his career at the University of Oxford. In 1993, Bob enthusiastically embraced a collaborative project to analyze complement evasion by *E. granulosus*, with Ana María Ferreira (Universidad de la República, Uruguay). In this context, Bob supervised the corresponding author’s doctoral Thesis, which was specifically aimed to find molecular mechanisms explaining poor host complement activation on the hydatid wall (Irigoín et al., 1996; Ferreira et al., 2001).

In the context of the Thesis mentioned, we detected an activity in hydatid wall extracts that inhibited the formation, and hence the activity, of the alternative complement pathway C3 convertase in assays using purified components (Díaz et al., 1999). This activity was independent of host factor H, which we had previously observed to be abundant in the same extracts (Díaz et al., 1997). Under the assay conditions, the novel activity prevented the proteolytic activation of factor B, an indispensable step in alternative pathway C3 convertase formation. It did not do so by inhibiting factor D, the proteinase that cleaves factor B. Hence the “inhibitor” had to block factor B’s binding to C3b (or, in our assay, amidated C3, which is a C3b analog), a step that is indispensable for factor B activation. The “inhibitor” was heat-stable, and behaved in gel permeation and dialysis as if it was a small macromolecule (Díaz et al., 1999).

The rest of the advances described in this Box were made mostly in Uruguay, but with Bob Sim’s important participation as senior collaborator and complement expert. The “inhibitor” was identified as inositol hexakiphosphate (Ins*p*_6_) (Irigoín et al., 2002). This compound has a high density of negative charge and therefore a very large hydrodynamic radius, explaining that it would behave like a macromolecule, though it is less than 1 kDa in molecular mass (Van der Kaay and Van Haastert, 1995). However, a difficult issue had surfaced: Ins*p*_6_ interacts strongly and in complex ways with divalent cations, which are needed in for complement activation. In particular, the alternative pathway C3 convertase is a kinetically unstable, Mg^2+^-dependent complex, the half-life of which is enhanced if Ni^2+^ is used instead of Mg^2+^ (Fishelson and Müller-Eberhard, 1982) as it had been done in our initial assays. We therefore needed two major inputs before we could fully interpret the results of our extensive set of functional complement assays. One was the detailed quantitative description of Ins*p*_6_-cation systems (Veiga et al., 2006; Torres et al., 2005). The second input was the verification that Ins*p*_6_ is found in the LL as its insoluble calcium salt (Irigoín et al., 2002, 2004; Casaravilla et al., 2006). Our hydatid wall extracts, prepared in the presence of the divalent metal sequestering agent EDTA, contained soluble Ins*p*_6_. In the presence of divalent cations, Ins*p*_6_ re-precipitated non-adverted to us (and to different extents depending on the cation). The inhibitory activity in our assays was not due to depletion of free divalent cations. Rather, the colloidal Ins*p*_6_ salts show specific interaction with factor B, in the presence of Ni^2+^ in particular, indeed preventing the factor B - C3b association (Irigoín et al., 2008). However, under the relevant combination of conditions, i.e. calcium Ins*p*_6_ and Mg^2+^ as the metal forming the convertase, only weak inhibition of complement activation is detected using purified components, and none in whole serum. In agreement, when human serum was put through a calcium Ins*p*_6_ column, factor B was not specifically retained. Instead, the SDS-PAGE profile of the eluate included the characteristic band pattern of C1q (Irigoín et al., 2008). The specific elimination of Ins*p*_6_ from the LL weakened the covalent deposition of C3b, suggesting that the calcium Ins*p*_6_ deposits acted as initiation sites for the classical complement pathway (Irigoín et al., 2008).

More recently, we determined that indeed, the native calcium Ins*p*_6_ deposits present in the LL bind C1q (and consequently C1 complex) avidly, and cause classical pathway initiation (Barrios et al., 2019). However, activation of C1 bound to Ins*p*_6_ is inefficient compared to activation on antibodies. Most of the C3b deposited downstream of this activation binds to calcium Ins*p*_6_ itself (Barrios et al., 2019), a phenomenon that was not detectable under the conditions of our previous assays (Irigoín et al., 2008). C3b deposited on calcium Ins*p*_6_ is extremely rapidly inactivated (Barrios et al., 2019), and this inactivation must depend on host factor H according to our early results (Díaz et al., 1997). Thus, we concluded that similar to other polyanions, calcium Ins*p*_6_ has, in addition to its strong affinity for C1q, some affinity for factor H (Barrios et al., 2019). Calcium Ins*p*_6_ probably contributes most sites for factor H sequestration on the LL [although the LL mucins likely also have sites as C3b inactivation on them is also rather fast (Irigoín et al., 2008)].

In sum, we began by studying factor H in the LL. We then found Ins*p*_6_ by following an activity on complement that turned out not to be biologically relevant. In the way, we observed that calcium Ins*p*_6_, the insoluble salt actually found in the LL, binds C1q and initiates classical pathway activation. However, this initiation does not make a net contribution to activation of the terminal complement pathway, partly because C3b on calcium Ins*p*_6_ is very efficiently inactivated. This in turn means that the calcium Ins*p*_6_ surface must has affinity for factor H, somehow taking us back to the beginning of the story. As explained in the main text, we now think that calcium Ins*p*_6_ acts a decoy target for the classical pathway that minimizes its antibody-dependent activation on the germinal layer (Barrios et al., 2019).

## Molecular immunological aspects: antibodies targeting the LL

The *E. granulosus* LL has abundant, strongly-bound host antibodies (Varela-Díaz and Coltorti, 1973; Coltorti and Varela-Díaz, 1974; Taherkhani et al., 2007; Riesle et al., 2014, Barrios et al., 2019). In human infection, these antibodies belong to IgG1, IgG2, IgG4, IgA, and IgE classes/sub-classes (Barrios et al., 2019). Most host antibodies remain bound after eliminating calcium Ins*p*_6_ with EDTA (Basika et al., 2012), suggesting that they are bound to the LL mucins. Although antibodies directed to the short non-glycosylated stretches of the LL predicted apomucins cannot be ruled out, in all likelihood an overwhelming majority of antibodies associating with the LL *in vivo* are bound to the mucin glycans. In agreement, *Echinococcus* infections prominently feature anti-carbohydrate and/or LL-reactive antibodies (Russi et al., 1974; Gottstein et al., 1994; Dai et al., 2001; Walker et al., 2004; Díaz et al., 2011b, 2016).

**Figure 3.**
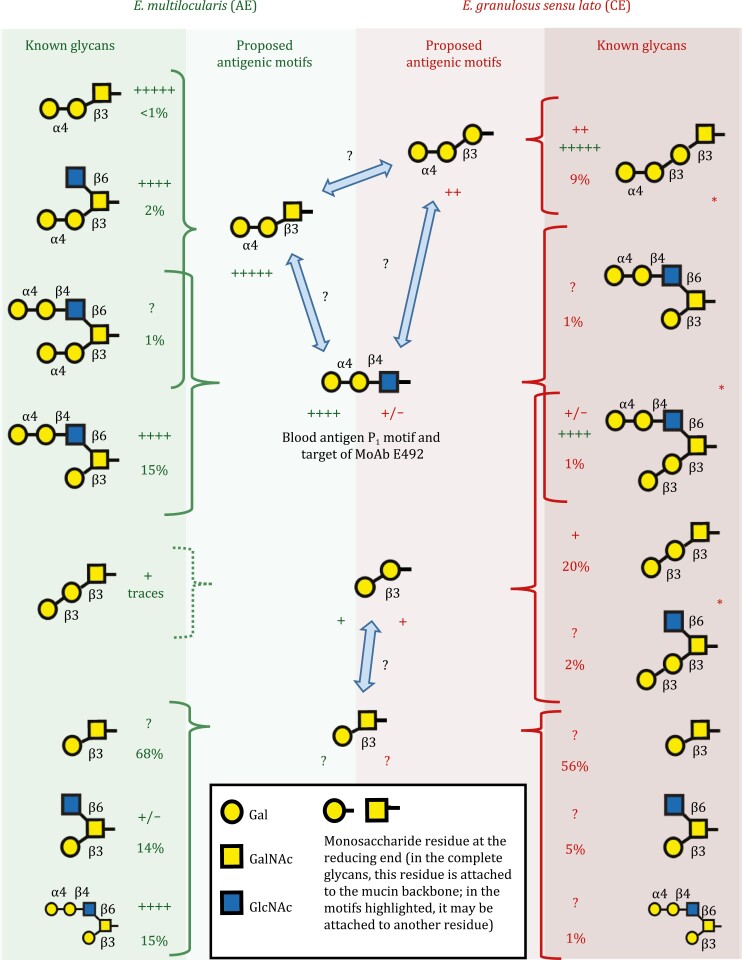
**Major carbohydrate antibody epitopes in the *E. multilocularis* and *E. granulosus* LL.** Information on *E. multilocularis* and *E. granulosus* is given in green and red font, respectively (and/or on green or red background). The main LL mucin glycans in each species (Díaz et al., 2009; Hulsmeier et al., 2010; Lin et al., 2013; del Puerto et al., 2016) are shown at either side of the figure. Antigenicity of the synthetic glycan against human infection sera (Koizumi et al., 2011; Yamano et al., 2012; Hada et al., 2021) (from +/‐ to +++++), and percent molar abundance in the LL (Díaz et al., 2009; del Puerto et al., 2016) are indicated for each structure. Glycans are grouped by the tri-saccharide or di-saccharide non-reducing terminal motifs that they contain, and some glycans contain more than one motif (a glycan that had to be represented twice for each species is shown in a smaller size in the second instances). For simplicity, branching at position 6 of GalNAc is taken not to abrogate the antigenicity of the motifs containing this sugar. Asterisks denote that, for *E. granulosus*, additional less abundant glycans exist that carry each of the motifs. The deduced antigenic motifs as such are shown in the center of the figure, with an indication of their inferred antigenicity in human infection. For some *E. granulosus* glycans that have not been detected in *E. multilocularis* (del Puerto et al., 2016), antigenicity towards *E. multilocularis* infection sera (Hada et al., 2021) is indicated in green. Such antigenicity may be explained by shared motifs or, in the absence of shared motifs, by cross-reaction with related motifs. The proposed cross-reactions between related motifs are indicated by light blue double arrows. Note that although no direct evidence exists for the motif Galβ1-3GalNAc being antigenic, the motif is highlighted because: (i) it is very abundant in the LL, and (ii) its possible antigenicity in *E. multilocularis* infection would explain why the related motif Galβ1-3Gal is antigenic in this species (Hada et al., 2021) in spite of being present only at trace levels (del Puerto et al., 2016). Also note that the glycan Galα1-4Galβ1-3Galβ1-3Galβ1-3GalNAc (not depicted) was not found to be antigenic against either *E. multilocularis* or *E. granulosus* infection sera (Hada et al., 2021), which does not fit the overall picture; we do not have an explanation for this observation.

The fine specificity of serum antibodies targeting the LL glycans has been mapped with the help of synthetic versions of those glycans (Koizumi et al., 2009). Human antibody responses to these synthetic glycans are generally stronger in AE than in CE (Hada et al., 2021). The immunodominant tri-saccharide motif in AE is Galα1-4Galβ1-3GalNAc (Koizumi et al., 2011; Yamano et al., 2012) (Fig. 3). The motif is present in its unmodified form in a glycan that is not abundant in the *E. multilocularis* LL (Hulsmeier et al., 2010; Díaz et al., 2015; del Puerto et al., 2016). However, glycans that carry variations of the same motif in which the GalNAc residue bears a GlcNAcβ1-6 branch are more abundant, antigenic, and probably cross-reactive with the unmodified motif (Hulsmeier et al., 2010; del Puerto et al., 2016; Hada et al., 2021). The Galα1-4Galβ1-4GlcNAc motif is also strongly antigenic in AE (Koizumi et al., 2011; Hada et al., 2021). In human CE, antibody responses against LL glycans appear to be dominated by motifs terminated in Galα, including Galα1-4Galβ1-3Gal (Díaz et al., 2011b; Yamano et al., 2012; Hada et al., 2021). However, the Galα1-4Galβ1-4GlcNAc motif appears not to be strongly antigenic in CE (Russi et al., 1974; Díaz et al., 2011b; Hada et al., 2021), in contrast to the situation in AE. In addition, antibody responses occur in CE against Galβ1-3Galβ1-3GalNAc (Hada et al., 2021). Although these responses are weak when measured against the synthetic glycan, they may be biologically significant as the glycan is very abundant in the *E. granulosus* LL (Díaz et al., 2009). The antigeniticy of the most abundant glycan in *E. granulosus* (Galβ1-3GalNAc) has not been measured. *E. multilocularis* infection antibodies are observed to react with glycans that are found only in the *E. granulosus* LL (Hada et al., 2021). This reactivity may be explained by cross-reactions, as also summarized in Fig. 3.

Three published mouse monoclonal antibodies are known to react with the *Echinococcus* LL. The E492 antibody (IgG) (Baz et al., 1999) reacts with the Galα1-4Galβ1-4GlcNAc motif (Lin et al., 2013) (Fig. 3). This motif, previously mentioned as a target of AE infection antibodies, is also responsible for the reactivity of human anti-P_1_ blood group antibodies with the LL (Russi et al., 1974). The E492 antibody reacts with the hydatid wall and with other structures both in *E. granulosus* and *E. multilocularis* (Baz et al., 1999; Walker et al., 2004), consistent with the wide occurrence of the target motif in the LL *O*-glycans, as well as in *N*-glycans and glycolipids found in other *Echinococcus* structures (Khoo et al., 1997; Lin et al., 2013; del Puerto et al., 2016; Díaz et al., 2016). The EmG3 antibody (IgM) also reacts strongly with the *E. granulosus* and *E. multilocularis* LL as well as with other *Echinococcus* structures (Grimm et al., 2020b; Reinehr et al., 2020). Its broad pattern of reactivity suggests that it may also recognize a carbohydrate epitope with broad distribution in *Echinococcus*, but its specificity is unknown. In contrast with the previous situations, the Em2G11 antibody (IgG) reacts with the *E. multilocularis* LL but not with the *E. granulosus* LL or with other *E. multilocularis* stuctures (Dai et al., 2001; Barth et al., 2012; Reinehr et al., 2020). The antibody binds carbohydrates (Dai et al., 2001), but it does not react with any of a number of synthetic glycans representative of the *E. multilocularis* LL glycans (Koizumi et al., 2011; del Puerto et al., 2016), and its specificity remains unknown.

## Shed LL materials

It is biological commonsense that the LL must turn over in order to allow the larval parasite to grow in size. In other words, the outermost strata of the LL, synthesized when the parasite was smaller, must be shed, and new strata incorporated from the inside. Given the massive size of the LL, this process has the potential to release considerable amounts of a parasite-derived “debris” within the host’s tissues. The accumulation and spread of such “debris” was first documented in *E. multilocularis* (Barth et al., 2012; Reinehr et al., 2020), and more recently in *E. granulosus* (Grimm et al., 2020a) infections in humans, and was achieved with the help of monoclonal antibodies Em2G11 and EmG3. The debris takes the form of particles given the names “SPEMs” and “SPEGs” (for “small particles of *E. multilocularis/granulosus*”), which measure 2–20 μm (in the parasite’s vicinity), or less than 1 μm (in the draining lymph nodes) (Barth et al., 2012; Grimm et al., 2020a, 2020b; Reinehr et al., 2020) (Thomas Barth, University of Ulm, personal communication). In the vicinity of the parasite, SPEMs/SPEGs are found in the histologically altered area close to the parasite and also in the surrounding vital host tissue (Grimm et al., 2020a, 2020b). The particles also reach the local blood vessels, and in liver they are found predominantly in the sinusoids, i.e. the vascular space. In *E. granulosus* infections in particular, particles were additionally found to be very abundant in a sample of pleural effusion (liquid escaping from the metacestode into the pleural space), as free particles and within macrophages. SPEMs/SPEGs also reach most of the regional lymph nodes in human infections (Grimm et al., 2020a). SPEMs/SPEGs have not yet been studied in natural or experimental animal infections.

The abundance of particulate LL material suggests the additional possibility of soluble LL-derived materials circulating in host fluids. Although this possibility has not yet been studied *in vivo*, *E. multilocularis* metacestodes in culture release soluble molecules recognized by the E492 antibody (Walker et al., 2004).

In sum, it can be expected that local host immune cells encounter the surface of the LL as such but much more commonly encounter LL-derived particles, both locally, and in draining lymph nodes. It will be important to characterize further the dissemination of LL-derived materials in infected hosts, including the soluble mucins, which might be detected with the help of the LL-reactive monoclonal antibodies available.

## Innate cell recognition of the LL: mechanisms dependent on Clec4F

The very abundant mucin glycans of the LL can be expected to engage host innate immunity via carbohydrate receptors (lectins), helping to shape host responses to larval *Echinococcus*. When the LL mucins were probed *in vitro* with a panel of recombinant lectin receptors expressed by mammalian innate immune cells, the only panel member to bind clearly was Clec4F (Hsu et al., 2013). Clec4F is a cell-surface receptor that has been studied almost exclusively in rodents, in which it is only expressed in Kupffer cells (Fadden et al., 2003; Yang et al., 2013; Taylor et al., 2019), the sessile liver macrophages exposed to the vascular space. Rodent Clec4F has specificity for glycans terminated in GalNAc or Gal (Fadden et al., 2003; Coombs et al., 2006; Yang et al., 2013; Taylor et al., 2019). This monosaccharide specificity is consistent with the LL mucin glycans being predominantly terminated in Gal (Díaz et al., 2009; Hulsmeier et al., 2010; Lin et al., 2013; del Puerto et al., 2016), and in fact several defined *E. granulosus* and/or *E. multilocularis* LL mucin glycans are bound by recombinant Clec4F *in vitro* (Hsu et al., 2013). We have recently obtained definitive evidence that mouse Kupffer cells take up *E. granulosus* LL mucins in Clec4F-dependent fashion *in vivo* (unpublished results) but the downstream consequences of this engagement/uptake are not known.

Rodent Clec4F participates in the clearance by Kupffer cells of platelets that have lost the terminal sialic acid residues from their surface glycans, thereby exposing sub-terminal Gal residues (Li et al., 2017; Jiang et al., 2021). Such a function is not expected to be associated to the generation of pro-inflammatory signals. Hence Clec4F matches the profile of a receptor that would be “chosen” through evolution by an immunity-evading parasite for binding its shed molecules and presumably helping in their clearance (Díaz and Allen, 2007). The liver-specific expression of Clec4F in rodents would also match the fact that the liver is the primary infection site of larval *E. multilocularis* in its rodent hosts. The liver was also probably the primary anatomical site for the larval stages of the ancestors of the genus *Echinococcus*, thought to infect rodents (Hoberg et al., 2000). In the natural hosts of larval *E. granulosus*, the situation is more complex: non-liver (particularly lung) infections are common (Thompson, 2017; Lightowlers et al., 2021), and Clec4F is probably expressed extra-hepatically in addition to in the liver (Taylor et al., 2019). In particular in sheep, which is the main host for *E. granulosus sensu stricto*, Clec4F is expressed (at the mRNA level at least) in liver, spleen, and lymph nodes (Taylor et al., 2019) (our unpublished observations). Importantly, Clec4F expressed in Kupffer cells may be relevant even if the parasite is not located in the liver, as shed LL materials can reach draining lymph nodes (Grimm et al., 2020a), and conceivably more distant anatomical sites including the liver.

The question of the relevance of Clec4F in human echinococcosis leads to a more complex discussion. Clec4F expression in liver (i.e. in Kupffer cells) has been lost in primates (Taylor et al., 2019). Further, in humans in particular, the Clec4F gene does not encode a functional lectin (Taylor et al., 2019). Importantly, mouse Clec4F appears not to function alone in the clearance of de-sialylated platelets by Kupffer cells. Rather, Clec4F seems to function as part of a system encompassing the asialoglycoprotein receptor (ASGR) and the macrophage Gal-type lectins (MGLs), also expressed in mouse Kupffer cells (Deppermann et al., 2020; Jiang et al., 2021), and having GalNac/Gal-type specificities (Coombs et al., 2006). In our reasoning, this opens the possibility that Kupffer cells in different mammalian species use different combinations of GalNac/Gal-type lectins to clear de-sialylated platelets. This in turn would mean that human Kupffer cells may express lectins that bind LL mucins even if they do not express functional Clec4F. In this respect, we did not detect clear-cut binding of recombinant (soluble, dimeric) human ASGR or MGL to LL mucins *in vitro* (Hsu et al., 2013), but this could not reflect the situation with the native (cell-surface, trimeric) receptors. If the ASGR does bind LL mucins, hepatocytes may contribute to the clearance of LL mucins (especially soluble), possibly in all host species as hepatocyte ASGR expression is conserved in mammals (Grewal, 2010). It should be borne in mind that humans are accidental hosts of larval *Echinococcus* parasites, and play no role in infection transmission. Hence, if lectin receptors are important for the handling of shed LL materials by the host, it is conceivable that such a mechanism is defective in humans.

In sum, Clec4F and possibly other lectin receptors of similar specificity probably contribute to the clearance of shed LL mucins by Kupffer cells and possibly further phagocytic cells. In terms of future perspectives, it will be important to characterize how the accumulation and circulation of LL materials is influenced by cellular capture mediated by Clec4F and possibly other lectins. A related point is the kinetics of digestion of LL-derived materials in host phagocytic cells including Kupffer cells. An additional point is whether the engagement of the host lectin receptors by LL materials generates intracellular signals beyond those needed for internalization. It will also be important to determine the identity and functionality of Clec4F-expressing cells present in lymphoid tissues of ungulate natural hosts of *E. granulosus*.

## Innate cell recognition of the LL: mechanisms independent of Clec4F

The pattern of Clec4F expression across cell types in the ruminant natural hosts of larval *E. granulosus* is not known. However, it is very likely at least some immune cells types that encounter LL particles *in vivo* will not express Clec4F. Focusing on dendritic cells (DC) and macrophages in particular, the most commonly used mouse cell models do not express Clec4F and are therefore suitable to study Clec4F-independent interactions with the LL. Such cell models include mouse bone marrow derived DC differentiated in the presence of GMCSF (GMCSF-BMDC) and bone marrow derived macrophages (BMDM). Both of these cell types do respond to *E. granulosus* LL particles, with up-regulation of CD86 (without concomitant up-regulation of CD40 or cytokine production) (Casaravilla et al., 2014). In the additional presence of TLR agonists (intended to mimic endogenous TLR agonists released *in vivo* as a consequence of tissue damage), the particles blunt CD40 up-regulation and IL-12 production while potentiating IL-10 production (Casaravilla et al., 2014). These responses to LL particles are not due to bound host antibodies (Casaravilla et al., 2014). They are also not significantly altered by removal of calcium Ins*p*_6_, by oxidation of the LL mucin glycans with periodate, or by catalytic hydrolysis of conventional proteins in the material (Casaravilla et al., 2014). However, reduction of disulfides in the material strongly diminishes the responses (Casaravilla et al., 2014; Pittini et al., 2019; Casaravilla et al., 2020). The single cysteine residue in the predicted LL apomucins is present in a non-glycosylated N-terminal sequence (Tsai et al., 2013): the corresponding synthetic peptide, dimerized via disulfide, does not imitate or competitively inhibit the responses elicited by LL particles (Pittini et al., 2019). As disulfide reduction weakens the LL mucin meshwork (Casaravilla and Díaz, 2010), an alternative possibility is that disulfides are needed for LL particles to maintain certain physical characteristics required for the cellular responses observed. This possibility is consistent with the observations that neither soluble nor solubilized and plate-bound LL mucins elicit responses similar to those triggered by the particles (Casaravilla et al., 2014; Pittini et al., 2019). In broad agreement, a soluble mucin fraction from the *E. multilocularis* LL does not elicit changes in surface markers in GMCSF-BMDC (Margos et al., 2011). So together the evidence suggests that the responses to LL particles depend on supra-molecular properties of the mucin gel, and not on conventional interactions between molecular-level agonistic motifs and cellular receptors.

Myeloid cells are thought to respond to particles by a mechanism independent of conventional cellular receptors, as studied for materials with adjuvant properties like alum, sodium urate, and polystyrene beads (Ng et al., 2008; Flach et al., 2011; Shi, 2012; Mu et al., 2018). This mechanism, termed “membrane affinity triggered signaling” (MATS) requires that the cells adhere to the particles strongly. Responses triggered via this mechanism are abrogated in the presence of inhibitors of actin dynamics or inhibitors of phosphatidylinositol 3-kinase (PI3K) class I (which acts in concert with actin filaments (Lindmo and Stenmark, 2006; Flach et al., 2011; Shi, 2012). Myeloid cell responses to LL particles were completely abrogated by inhibitors of actin dynamics and of PI3K class I, in agreement with a MATS-like mechanism (Casaravilla et al., 2014; Pittini et al., 2019; Casaravilla et al., 2020). In further agreement, responses to particulate adjuvants thought to act by MATS can take place in the absence of particle internalization (Ng et al., 2008; Flach et al., 2011) and LL particles of sizes far too large for cellular internalization elicit responses similarly to smaller particles (Pittini et al., 2019; Casaravilla et al., 2020). Particulate adjuvants thought to act via MATS activate the NLRP3 inflammasome in DC primed with TLR adjuvants *in vitro*, or without need of such priming *in vivo* (Kool et al., 2008; Eisenbarth et al., 2008; Khameneh et al., 2017). The same activity is shown by LL particles on GMCSF-BMDC, again dependent on actin dynamics and PI3K class I but not on particle internalization (Casaravilla et al., 2020). NLRP3-dependent IL-1β, indicative of NLRP3 inflammasome activation is also detected *in vivo* after particle injection into the peritoneal cavity (Casaravilla et al., 2020). Thus, we see a MATS-like mechanism as a reasonable explanation for the capacity of LL particles to elicit responses in myeloid cells in a manner not dependent on any particular molecular-level motifs but instead dependent on their physical presentation. It is noteworthy that particles previously proposed to act via MATS are rigid in nature (crystals, polystyrene beads), whereas LL mucin particles are based on an aqueous gel and hence soft and flexible.

Exposure to LL particles does not cause activation of the central signaling module NF-κB or MAP kinases, or interfere with their activation in response to LPS, in GMCSF-BMDC. However, it does blunt the activation of Akt, an effector of PI3K class I, induced by LPS (Pittini et al., 2019). Similarly muted Akt activation is observed in response to disparate signals capable of activating PI3K class I, in GMCSF-BMDC and inflammatory macrophages exposed to LL particles (Seoane et al., 2016). This may seem hard to reconcile with PI3K class I being absolutely required for cells to respond to the particles. However, it should be first noted that Akt activation is blunted but not fully inhibited in the presence of the particles, and presumably the same happens with PI3K upstream of Akt. Moreover, an attractive explanatory hypothesis can be put forward based on details of the MATS mechanism. The interaction between the DC plasma membrane and polystyrene beads causes local accumulation of the lipid phosphatidylinositol 4,5-bisphosphate (PIP2) in the inner leaflet of the plasma membrane (Mu et al., 2018). This brings about recruitment of the cytosolic protein moesin and exposure of an atypical ITAM sequence that initiates MATS signaling. PIP2 is the substrate of PI3K class I (Hawkins and Stephens, 2015), and PIP2 availability can regulate the enzyme’s activity in some contexts (Saito et al., 2003; Schwartzberg, 2003). We envisage that PIP2 may be strongly accumulated in the plasma membrane area in contact with LL particles, and that this may result in diminished availability of PIP2 for agonist-induced PI3K class I activity, and hence in blunted Akt activation. In our hypothesis, PIP2 sequestration would take place to a larger extent during interaction with LL particles than it does during interaction with alum in particular, since alum does not blunt Akt activation in GMCSF-BMDC (Casaravilla et al., 2020).

**Figure 4.**
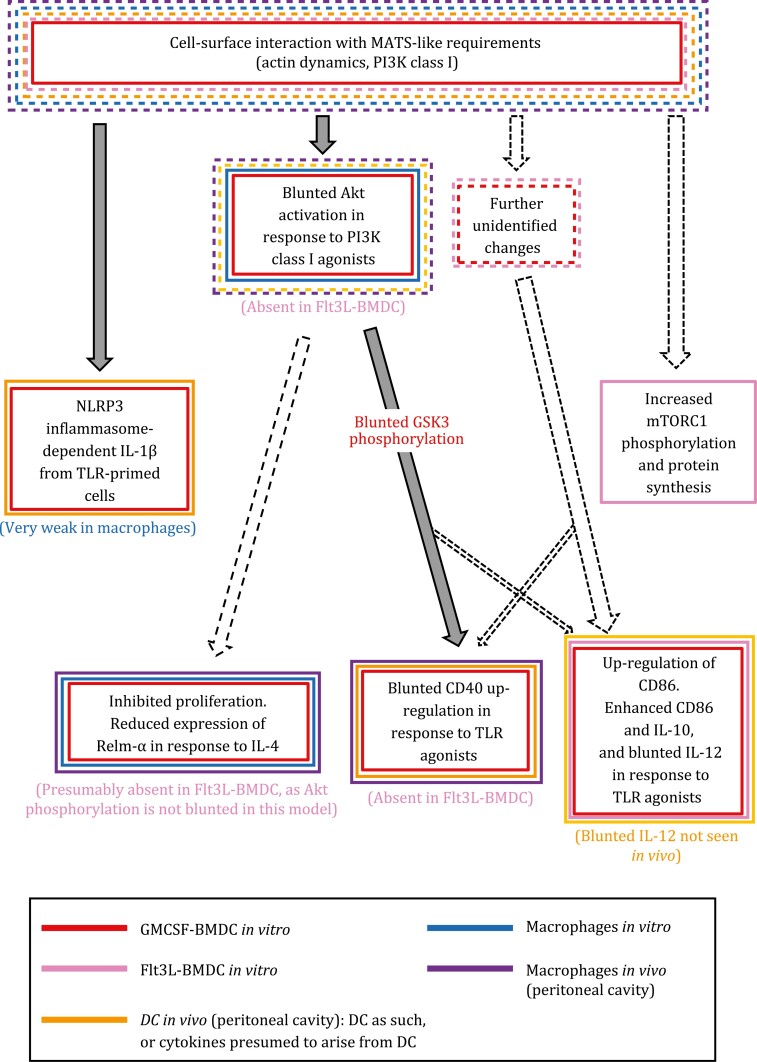
Summary of proposed responses to LL particles observed in DC and macrophages, excluding any lectin receptor-dependent responses. The diagram attempts to summarize responses tentatively attributed to a MATS-like mechanism. The solid color-coded rectangles indicate the cell models in which there is supporting evidence for the corresponding event. The dotted rectangles, which have the same color code, indicate that the event is deduced from context to take place in the particular cell model. Grey arrows indicate mechanistic evidence, whereas empty dotted arrows indicate deduced mechanistic links, narrow arrows indicating weak possible links. The diagram is based on references (Casaravilla et al., 2014; Seoane et al., 2016; Pittini et al., 2019; Casaravilla et al., 2020; Rodrigues et al., 2021). The *in vitro* macrophage data correspond to BMDM or thioglycollate medium-elicited peritoneal cavity macrophages. The alterations in CD40, CD86, IL-10, and IL-12 in GMCSF-BMDC are known to be independent of NLRP3 and of PI3K class III (needed for NLRP3 inflammasome activation by LL particles) (Casaravilla et al., 2020). Although not shown in the figure, the mTORC1 complex is (indirectly) activated by Akt (in addition to other pathways). Thus, enhanced mTORC1 activation after exposure to LL particles, observed in Flt3L-BMDC (Rodrigues et al., 2021), is unlikely to occur in GMCSF-BMDC and macrophages, which respond to the particles with decreased capacity to activate Akt in response to PI3K class I agonists (Seoane et al., 2016; Pittini et al., 2019).

Blunted Akt activation in response to LL particles results in diminished phosphorylation of Akt’s direct target GSK3, and these changes explain that GMCSF-BMDC exposed to the particles do not up-regulate CD40 normally in response to TLR agonists (Casaravilla et al., 2014; Pittini et al., 2019) (Fig. 4). Slightly blunted CD40 up-regulation in the presence of LL particles is also seen in peritoneal cavity DC *in vivo* (Casaravilla et al., 2014). The interaction between CD40 and its ligand CD154 is a central activating signal in the immune system, and is required for induction of most CD4^+^ T-cell responses by DC (Díaz et al., 2021).

As mentioned, LL particles diminish Akt activation and blunt CD40 up-regulation in GMCSF-BMDC. However, a different picture is seen in experiments using BMDC differentiated in the presence of Flt3L (Flt3L-BMDC). These are a model for splenic (and likely other resident) DC, whereas GMCSF-BMDC are a model for inflammatory monocyte-derived DC (Naik, 2008). Flt3L-BMDC do not show reduced Akt or GSK3 phosphorylation in the presence of LL particles (Pittini et al., 2019). They also do not show muted CD40 up-regulation, and in fact they show the opposite trend (Pittini et al., 2019). One of the major downstream targets indirectly activated by Akt is the mTORC1 complex (Manning and Toker, 2017). Flt3L-BMDC are reported to respond to LL particles with enhanced mTOR phosphorylation and overall protein synthesis rate, suggestive of increased activation of the mTORC1 complex (Rodrigues et al., 2021). These observations strongly suggest that different DC sub-types can respond differently to LL particles (Fig. 4). Somewhat similarly, Flt3L-BMDC, but not GMCSF-BMDC, up-regulate CD40 in response to *Schistosoma mansoni* soluble egg antigen (Webb et al., 2017). Interestingly, CD40 up-regulation in this context depends on a type I interferon response by the DC themselves, which is necessary for induction of CD4^+^ T-cell responses to the parasite preparation.

LL-derived materials, particulate, or soluble, may also influence myeloid cells through non-intrinsic proteins that become adsorbed onto the structural mucins and in particular onto calcium Ins*p*_6_ (Basika et al., 2012). We speculate that adsorbed protein explains the induction of arginase (a marker of macrophage of M(IL-4) phenotype (Rückerl and Allen, 2014)) and inhibition of nitric oxide production in macrophages by soluble LL extracts previously observed (Amri and Touil-Boukoffa, 2015). We wash the materials extensively with high ionic strength to remove adsorbed proteins and do not observe LL-induced arginase or M(IL-4) markers in macrophages *in vitro* (Díaz et al., 2016; Seoane et al., 2016). Arginase induction in the cited paper (Amri and Touil-Boukoffa, 2015) was abrogated by mannan, suggesting participation of the mannose receptor, known to induce arginase expression in macrophages in some contexts (Garrido et al., 2011; Nzoumbou et al., 2017). Potential mannose receptor ligands extracts are high mannose *N*-glycans (Khoo et al., 1997; Ezekowitz et al., 1988) probably derived from adsorbed glycoproteins, as *N*-glycans altogether are not intrinsic constituents of the LL (Díaz et al., 2009).

In sum myeloid cell types not expressing Clec4F respond to LL particles by mechanisms that appear to be receptor-independent and depend on physical properties of the particles. These mechanisms are akin to those explaining responses to particulate adjuvants, but certain differences with responses to adjuvants are apparent.

In terms of future perspectives, further research would be worthwhile on Flt3L-BMDC responses to LL particles, including assessing if there is a type I interferon response similar to that observed for *S. mansoni* soluble egg antigen (Webb et al., 2017). Flt3L-BMDC include both conventional and plasmacytoid DC (Naik et al., 2005): the analysis of plasmacytoid DC responses to LL materials holds particular interest, as this cell type has been recently found to be enriched near the parasite in human cystic echinococcosis (Yasen et al., 2021). In addition, the study of myeloid cell responses to LL particles should incorporate a systematic analysis of the influence of host proteins adsorbed *in vivo*, using homologous (e.g. mouse-mouse) systems. Experiments with myeloid cells should also address further whether LL particles induce the expression of central regulatory molecules such as TGF-β, PD-L1, and PD-L2.

## Impact of laminated layer materials on immune biology of the echinococcoses: interactions with response induction

Adaptive immune responses, including T-cell responses, are readily detectable in *Echinococcus* infections (Wang and Gottstein, 2016; Díaz, 2017; Gottstein et al., 2017). The phenotype of DC in the parasite’s vicinity has been analyzed in experimental intraperitoneal *E. multilocularis* infection. The cells (defined only in terms of CD11c^+^ expression) were found to express lower protein levels of CD40, CD80, CD86, and MHCII than cells from naïve mice (Mejri et al., 2011). They were also found to have augmented levels of mRNA for TGF-β, and to suppress mitogen-induced T-cell proliferation *ex-vivo*. This is broadly consistent with the previously mentioned importance of regulatory T-cells in responses to larval *Echinococcus*.

The histological data on SPEMs/SPEGs discussed above indicate that extracellular LL particles must contact DCs both at the tissue site and in draining lymph nodes. The available data do not allow a safe inference on the impact of such conditioning on the polarization of the subsequent adaptive response. Some characteristics of GMCSF-BMDC conditioned by *E. granulosus* LL particles (low CD40, high IL-10, and low IL-12 in the additional presence of TLR agonists) suggest a tolerogenic profile. However, other characteristics (higher than basal CD86, IL-1β secretion in the presence of TLR agonists) suggest the opposite (Casaravilla et al., 2014; Casaravilla et al., 2020). Also, as mentioned, Flt3L-BMDC conditioned by LL particles up-regulate CD40 normally, unlike GMCSF-BMDC (Pittini et al., 2019). Injection of LL particles triggers antigen-specific IL-13 and IL-10 responses, without detectable IL-5 or interferon-γ, suggesting a regulatory/Th2 bias (our unpublished results). Our group is currently analyzing the potential of LL particles to expand regulatory T cells *in vivo*, with promising results.

For LL particles to be a source of antigens for T-cell-dependent responses, DC must phagocytose the particles, at the tissue site and/or in lymph nodes. Although we have not observed phagocytosis *in vitro* (Pittini et al., 2019), opsonization with antibodies and/or complement, which likely takes place *in vivo*, has not been assessed. Such opsonization could involve natural anti-carbohydrate antibodies (Milland and Sandrin, 2006) in addition to antibodies elicited by the infection. The overall capacity to internalize antigens is not inhibited in DC exposed to LL particles, as Flt3L-BMDC conditioned with the particles endocytose ovalbumin *in vitro* (Rodrigues et al., 2021), and similarly conditioned GMCSF-BMDC phagocytose plastic beads (our unpublished data). GMCSF-BMDC conditioned with LL particles are functional in terms of antigen presentation, as (once pulsed with ovalbumin peptide) they efficiently induce the proliferation of ovalbumin-specific CD4^+^ T cells *in vitro* (Seoane et al., 2016). As mentioned, injection of LL particles triggers detectable antigen-specific responses, which implies that the particles are taken up and processed by antigen-presenting cells *in vivo* (our unpublished results). In addition to particles, DC could incorporate soluble LL mucins: a soluble *E. multilocularis* LL fraction was shown to be taken up by resident mouse peritoneal cells *in vitro* (Dai et al., 2001).

Within lymph nodes in human infections, SPEGs are found both in the sinuses and in the germinal centers, i.e. the sites in which B cells are stimulated with the collaboration of T cells. Within germinal centers, the particles co-localize with follicular dendritic cells (FDC). FDC are cells specialized in the long-term retention of antigens opsonized by antibody and/or complement for the stimulation of B cells differentiating in the germinal center (Rezk et al., 2013). Co-localization with FDC suggests that LL particles effectively become opsonized in the infection context. Consistently, the mechanisms of complement activation and control outlined above imply that the LL becomes opsonized with iC3b and its downstream product C3dg, for which FDC express the receptor CR2 (Carroll and Isenman, 2012). Lymph nodes that contain SPEMs/SPEGs are statistically larger than patients’ lymph nodes that do not contain these particles, as well as larger than lymph nodes from control individuals (Grimm et al., 2020a), suggesting ongoing immune responses. Although these data do not imply causal relationships, they do suggest that SPEMs/SPEGs are a probable source of antigens for T-cell dependent B-cell responses, which again implies that DC internalize the particles *in vivo*. Consistent with our proposal that the LL mucins are poor in T-cell epitopes (Díaz et al., 2011b), neither of two different purified mucin fractions from the *E. multilocularis* LL elicits proliferation of spleen cells from infected mice *in vitro*, in contrast to preparations that contain conventional parasite antigens (Dai et al., 2001; Walker et al., 2004; Margos et al., 2011). According to our unpublished proteomic studies, it is unlikely that the (*E. granulosus*) LL structure includes conventional (non-mucin) proteins. We reason that an alternative source of T-cell antigens present in SPEMs/SPEGs *in vivo* are conventional parasite proteins (potentially including the major diagnostic antigens (Díaz et al., 2016) adsorbed onto the structural LL components. As mentioned, in *E. granulosus*, calcium Ins*p*_6_ can adsorb conventional proteins (Casaravilla et al., 2006; Basika et al., 2012). In experiments in which LL-derived from natural infections is injected into mice (as in our own experiments mentioned in the previous paragraphs), strongly-bound proteins from the natural host may also act as T cell antigens.

SPEMs/SPEGs may also collaborate towards T-cell independent B-cell responses, as previously suggested (Díaz et al., 2011b). Antibodies induced in experimental *E. multilocularis* infection reactive with a LL mucin fraction are mostly T cell-independent (Dai et al., 2001). The induction of T-independent, LL-reactive antibodies may require priming by cross-reactive, non-LL, components exposed by the infecting parasite stages: in experimental *E. multilocularis* infection, LL-reactive antibodies are observed when mice are infected with protoscoleces i.p. but not when they are infected with eggs orally (Walker et al., 2004). Protoscoleces (from *E. granulosus*) are known to contain glycoconjugates that react with the antibody E492 (also reactive with the hydatid wall, as mentioned) that elicit T-cell independent antibodies after their injection in mice (Mourglia-Ettlin et al., 2011).

Capture of LL materials by the liver, and by Kupffer cells in particular, could be a major player influencing adaptive immune response induction in *Echinococcus* infections. Such capture could limit the amount of LL materials that reaches the spleen. In addition, such capture might generate parasite-specific regulatory responses that temper effector adaptive responses against the parasite. The liver (under non-inflammatory conditions) carries out extra-lymphoid tolerogenic antigen presentation, which can impact at a systemic level (Lüth et al., 2008; Thomson and Knolle, 2010; Heymann and Tacke, 2016). Kupffer cells in particular play a major role in tolerogenic antigen presentation to CD4^+^ T cells (You et al., 2008; Heymann et al., 2015). Although nothing is known about the cells present in lymphoid tissues of non-rodent non-primate mammals that express Clec4F (Taylor et al., 2019), it is plausible that these cells may also contribute to induction of tolerance to parasite antigens.

In broader terms, the presence of molecules with potential to induce regulatory responses in the LL would also be suggested by the effects of a soluble extract on whole spleen cells stimulated *in vitro* with LPS: in cells treated with the extract, transcription of Th1-inflammatory molecules (IFN-γ, IL-1β, TNF-α) was decreased whereas transcription of regulatory markers (TGF-β and Foxp3) was increased (Benazzouz et al., 2021). It should however be cautioned that the mentioned extract is likely to contain host (human) proteins as major components. Also, a mucin fraction from the *E. multilocularis* LL strongly suppresses spleen cell proliferation induced by conventional parasite antigens or mitogen (Walker et al., 2004). On the other hand, injection of LL particles in the absence of another challenge is reported to elicit inflammatory cytokines, suggesting that they have an inflammatory potential (Shakibapour et al., 2020; Benazzouz et al., 2021).

In sum, we lack direct information on what is the impact of DC conditioning by LL materials on subsequent adaptive responses. It is likely that LL particles have an inflammatory potential that is associated with their physical presentation: disparate particulate materials have inflammatory and adjuvant properties (Harris et al., 2010; Maughan et al., 2015). However, in the case of the LL this inflammatory potential has probably been curtailed to a minimum through evolution. The limitation by LL particles of Akt activation on inflammatory DC models and its downstream effects (Seoane et al., 2016; Pittini et al., 2019) may reflect this adaptation. DC and other conventional antigen-presenting cells must be able to take up LL particles *in vivo*, presumably with the help of opsonization by antibody and/or complement. In terms of the LL as a source of antigens, it is unclear if the LL mucins can provide T cell epitopes, and whether these mucins can elicit, or only amplify, T-independent B-cell responses. However, conventional antigens are probably taken up together with the mucin particles. The lectin-mediated uptake of LL materials by Kupffer cells may be an important player, by limiting the availability of these materials for interaction with conventional antigen-presenting cells and lymphocytes and/or by inducing regulatory responses to parasite antigens.

## Impact of laminated layer materials on the immune biology of the echinococcoses: interactions with effector mechanisms

The LL operates as a barrier against direct contact between parasite cells and host immune cells. However, this does not offer complete protection to the parasite. *In vitro*, nitric oxide can diffuse through the LL and kill *E. granulosus* larvae (Steers et al., 2001). *In vivo* in natural infections, local granulomatous inflammation strongly correlates with low parasite viability (Díaz et al., 2018). The previous discussion on complement suggests that the *E. granulosus* LL has been perfected through evolution for minimal generation of complement-derived pro-inflammatory factors. In agreement, early in secondary *E. granulosus* experimental infections, surviving parasites show little inflammation and complement deposition on the newly formed LL; in contrast, those parasites that are killed before deploying a LL show complement deposition on their surface and are surrounded by inflammatory cells (Breijo et al., 2008).

In natural infections by larval *Echinococcus*, local inflammation when present can be eosinophil-rich, but it most often features macrophages and macrophage-derived cells types as central effector cells (Díaz et al., 2018). In experimental intra-hepatic *E. multilocularis* infection, depletion of macrophages during the first 6 weeks facilitates parasite establishment and perhaps also parasite development after LL deployment (Wang et al., 2020). We will therefore focus on macrophages in the following discussion.

Macrophage accumulation at infection sites can obviously be brought about by recruitment of their monocyte precursors. However, macrophage accumulation can also be caused by macrophage proliferation at the site, and this process is marked in type 2 contexts such as helminth infections (Rückerl and Allen, 2014; Jenkins and Allen, 2021). Such supra-homeostatic macrophage proliferation can be induced by the trophic factor that maintains macrophages numbers under basal conditions (M-CSF or CSF1) or by type 2 cytokines including IL-4 (Rückerl and Allen, 2014; Jenkins and Allen, 2021). The contributions of macrophage recruitment and proliferation to macrophage accumulation in chronic *Echinococcus* infections have not been assessed.

LL particles at low doses inhibit macrophage proliferation induced by exogenous M-CSF or IL-4, apparently as a consequence of their capacity to inhibit PI3K/Akt signaling induced by these agonists (Seoane et al., 2016). This activity has the potential to curtail macrophage accumulation in the vicinity of the parasite (given that IL-4 is present among host responses in the echinococcoses (Ma et al., 2014; Pang et al., 2014a, 2014b; Wang et al., 2014; Petrone et al., 2015), thus in principle helping parasite survival. However, higher doses of LL particles also cause recruitment of monocytes that differentiate into macrophages (Seoane et al., 2016) (our unpublished results).

Macrophages are highly plastic cells, and as such capable of being anti-parasite effectors as well as of suppressing effector responses (Ruckerl et al. 2014). The major recognized macrophage polarization program in type 2 contexts is the M(IL-4) phenotype, so termed because the major responsible cytokine is IL-4 (Ruckerl et al. 2014). Strong macrophage M(IL-4) differentiation takes place in experimental intra-hepatic *E. multilocularis* infection, both before, and after LL deployment (Wang et al., 2020). Also, depletion of macrophages in this system decreases local fibrosis, in agreement with M(IL-4) macrophages being associated with fibrosis in other systems (Ruckerl et al. 2014). Although as mentioned depletion of macrophages favors parasite establishment/development in this system, an-anti-parasitic role for M(IL-4) macrophages should not be inferred, as there is also an early wave of “classically activated” macrophages that may contribute to the result (Wang et al., 2020). The enzyme arginase, an M(IL-4) marker (Rückerl and Allen, 2014), is upregulated in macrophages and other local cells in experimental intraperitoneal *E. granulosus sensu stricto* infection (Cao et al., 2020). This apparently results in a reduction in circulating levels of arginine and consequent decrease in CD3ζ expression in T cells (Cao et al., 2020), as reported in other systems (Rodriguez et al., 2003).

The effects of LL materials on M(IL-4) phenotypes appear to be complex. LL particles *in vitro* and at low doses *in vivo* inhibit the induction (by exogenous IL-4) of the M(IL-4) marker Relm-α but not that of ChiL3 (also known as Ym1). This is also probably explained by the blunting of PI3K class I/Akt activation, as induction of Relm-α depends heavily on this pathway whereas induction of ChiL3 does not (Heller et al., 2008; Weisser et al., 2011; Rückerl et al., 2012; Byles et al., 2013; Covarrubias et al., 2016). However, at high doses *in vivo*, LL particles cause up-regulation of Relm-α, ChiL3, and arginase ((Seoane et al., 2016); our unpublished results).

Helminth immune evasion probably rests mostly on the regulatory, rather than on type 2, arm of responses (Díaz and Allen, 2007). As mentioned, evidence is growing for strong regulatory responses in the echinococcoses (Mejri et al., 2011; Pang et al., 2014a, 2014b; Gottstein et al., 2017; Díaz et al., 2018; Pang et al., 2018; Wang et al., 2018; Fratini et al., 2020). In macrophages in particular, M(IL-4) markers can co-exist with different levels of expression of immune-stimulatory (e.g. CD40, CD80, CD86) and immune-inhibitory molecules (e.g. PD-L1, PD-L2), and these can differ across macrophage sub-types at a given infection site (Campbell et al., 2018). In experimental intraperitoneal *E. multilocularis* infection, local macrophages express levels of CD80/CD86 similar to those in naïve mice and levels of CD40 below those of naïve mice (Mejri et al., 2006). Macrophages from the infected mice are poorer than those from naïve mice at stimulating antigen-specific T-cell proliferation, and they suppress T-cell proliferation induced by a mitogen. In the mitogen system, an agonistic CD40 antibody potentiates the T-cell stimulatory capacity of macrophages from naïve mice but not of those from infected mice, suggesting defective capacity to respond via CD40 (Mejri and Gottstein, 2006). Decreased CD40 up-regulation (in the presence of TLR agonists *in vitro*) is observed in BMDM exposed to *E. granulosus* LL particles (Casaravilla et al., 2014). Speculatively, contact of macrophages with SPEGs/SPEMs *in vivo* may decrease their CD40 expression and capacity to interact with CD4^+^ T cells, thus curtailing macrophage effector responses that require T cell help. Also, negative impacts of *E. granulosus* and *E. multilocularis* LL materials on macrophage effector functions is suggested by *in vitro* experiments analyzing nitric oxide production (Steers et al., 2001; Andrade et al., 2004).

More broadly, a potential of LL materials to blunt effector responses is suggested by major amelioration of dextran sulfate-induced colitis and reduction in plasma TNF-α and IFN-γ in mice injected i.p. with LL particles (Soufli et al., 2015).

In sum, the LL as such acts a barrier against cell-mediated effector mechanisms and it is evolutionarily optimized to minimize complement-dependent cell recruitment. LL particles can inhibit macrophage accumulation by proliferation. Whereas it is clear that LL materials can antagonize classical macrophage effector functions and suppress some responses via arginase, data are still missing on the capacity of LL materials to induce expression of (non-Th2) immune-inhibitory molecules, in macrophages, and other cell types.

## Conclusions

The LL primarily fulfills a physical defensive function for larval *Echinococcus* parasites. The growth of these larvae requires the LL to be turned over, generating debris in the host tissues and in draining lymph nodes. There must have been strong evolutionary pressure for this debris to avoid triggering inflammatory responses that, considering its sheer abundance, would endanger the host’s life. This pressure is reflected in the subtle interactions with complement, which we now understand to a reasonable degree for *E. granulosus*. Since attaining an overall lack of pro-inflammatory signals often requires active downregulation, *Echinococcus* may have developed the capacity to deliver anti-inflammatory signals to host immune cells via LL materials. Despite some interesting hints, the search for such signals has proven hard. A major difficulty is that particulate materials have immunological activities by virtue of being particulate, making it impossible to choose an “inert” particulate material that acts as a point of comparison. The LL mucin glycans engage host Clec4F, expressed in most mammalian Kupffer cells, which surely provides a means for handling of LL materials by the host. Finally, it is obvious that host responses to LL materials must be shaped by parasite-derived immune regulators unrelated to the LL. It is crucial to characterize such immune regulators, and the excretion-secretion products of metacestodes are the main starting point (Nono et al., 2012; Nono et al., 2020).

## Abbreviations

ACP: alternative complement pathway
AE: alveolar echinococcosis
ASGR: asialoglycoprotein receptor
BMDC: bone marrow derived DC
BMDM: bone marrow derived macrophages
CCP: classical complement pathway
CE: cystic echinococcosis
DC: dendritic cell(s)
FDC: follicular dendritic cells
Flt3L-BMDC: BMDC differentiated in the presence of Flt3L
Gal: galactose
GalNAc: *N*-acetylgalactosamine
GlcNAc: *N*-acetylglucosamine
GMCSF-BMDC: BMDC differentiated in the presence of GMCSF
Ins*p*_6_: *myo*-inositol hexakisphosphate
LL: laminated layer
MATS: membrane affinity triggered signaling
MGL: macrophage galactose-type lectin
SPEGs: small particles of *Echinococcus granulosus*
SPEMs: small particles of *Echinococcus multilocularis*

## Declarations

This work was supported by Agencia Nacional de Investigación e Innovación, Government of Uruguay (ANII) (grant FCE_1_2019_1_156234; to author CC) and a by Comisión Sectorial de Investigación Científica, Universidad de la República, Uruguay (“CSIC Iniciación” grant id 575 year 2019; to author LG).

In memory of Bob Sim. This article is dedicated to the memory of Robert B. (“Bob”) Sim, who passed away in 2021. Bob was a world authority in complement biochemistry and an outstanding scientific mentor. Part of the advances summarized in this article were made possible by Bob’s direct contribution, his inspirational supervision of the article’s corresponding author, and his generous advice and help with reagents.
